# CLINICIAN INTERACTIONS WITH OLDER LGBT+ PATIENTS IN COLORADO: VISIBILITY, IDENTITY, AND EQUITABLE CARE

**DOI:** 10.1093/geroni/igae098.0796

**Published:** 2024-12-31

**Authors:** Korijna Valenti

**Affiliations:** University of Alabama at Birmingham, Birmingham, Alabama, United States

## Abstract

The unique needs of older lesbian, gay, bisexual, transgender (LGBT+) patients continue to merit attention. It is critical to fill the gap between clinician-patient communication, viewing patients as individuals with distinct support systems, and spouse/partner inclusivity. Guided by a queer gerontology framework, this study analyzed perspectives of clinicians across Colorado (n = 20) regarding their experiences interacting with LGBT+ patients 55+ with serious illness, including a) how health care professionals perceive their knowledge of and interactions with LGBTQ+ patients; (b) how these perspectives may inform patient care; and (c) how to best conceptualize communication between clinicians and LGBTQ+ patients. Inductive thematic analysis revealed three main themes: 1) visibility and treatment of LGBTQ+ patients, 2) asking about identity, and 3) acknowledging limitations around the current state of care for LGBTQ+ patients. Participants noted varying levels of comfort with and knowledge about LGBTQ+ patients and patients’ chosen care partners Participants addressed barriers around not asking about sexual orientation or gender identity, treating all patients “the same,” and the need for increased advocacy and proactive communication by health care professionals. Findings reveal the need for improved knowledge acquisition, training, and inclusivity for clinicians to fully address older LGBT+ patient needs. Several participants noted experiences around discrimination in the clinical environment and the need to “do better,” learn more, and engage more deeply with patients. Clinician understanding of patient needs, the use of correct language, and familiarity with patients’ chosen care partners may lead to improved health care experiences for LGBT+ patients.

